# M6A RNA methylation in biliary tract cancer: the function roles and potential therapeutic implications

**DOI:** 10.1038/s41420-024-01849-z

**Published:** 2024-02-16

**Authors:** Xuesong Bai, Jianhao Huang, Yiqun Jin, Jiemin Chen, Shengnan Zhou, Liangbo Dong, Xianlin Han, Xiaodong He

**Affiliations:** 1 Department of General Surgery, Peking Union Medical College Hospital, Peking Union Medical College & Chinese Academy of Medical Sciences, Beijing, China; 2 Department of Ultrasound, Affiliated Hangzhou First People’s Hospital, School Of Medicine, Westlake University, Hangzhou, China; 3 Department of Gastroenterology, Peking Union Medical College Hospital, Peking Union Medical College & Chinese Academy of Medical Sciences, Beijing, China; 4 Department of Gastrointestinal Surgery, China-Japan Friendship Hospital, Beijing, China

**Keywords:** Biological sciences, Biliary tract cancer, Epigenetics

## Abstract

Biliary tract cancers (BTCs) are relatively rare malignancies with a poor prognosis. For advanced BTCs, the efficacy of current chemotherapeutic approaches is limited. Consequently, there is an urgent need to deepen our understanding of the molecular mechanisms underlying BTC tumorigenesis and development for the exploration of effective targeted therapies. N6-methyladenosine (m6A), the most abundant RNA modifications in eukaryotes, is found usually dysregulated and involved in tumorigenesis, progression, and drug resistance in tumors. Numerous studies have confirmed that aberrant m6A regulators function as either oncogenes or tumor suppressors in BTCs by the reversible regulation of RNA metabolism, including splicing, export, degradation and translation. In this review, we summarized the current roles of the m6A regulators and their functional impacts on RNA fate in BTCs. The improved understanding of m6A modification in BTCs also provides a reasonable outlook for the exploration of new diagnostic strategies and efficient therapeutic targets.

## Facts

- M6A modification plays important roles in regulating RNA metabolism in biliary tract cancers.
- M6A regulators function as tumor suppressors or oncogenes in carcinogenesis and progression of biliary tract cancers.
- M6A modification shows significance in clinical diagnosis and treatment of biliary tract cancers, and targeting m6A regulators presents a captivating and promising prospect in cancer treatment.

## Open questions

- What are specific molecular mechanisms by which m6A modification affects the tumorigenesis and development of biliary tract cancers?
- Which m6A-related molecules can serve as biomarkers for the diagnosis and prognosis of biliary tract cancers?
- Whether FTO inhibitors could be applied to biliary tract cancers, particularly in cases with IDH1/2 mutations?
- How to improve the sensitivity of drug treatments for biliary tract cancers, through the regulation on m6A modifications?

## Introduction

Biliary tract cancer (BTC) is a heterogeneous spectrum of malignancies including cholangiocarcinoma (intrahepatic/extrahepatic cholangiocarcinoma, iCCA/eCCA), gallbladder cancer (GBC), and ampullary cancer, with a 5-year overall survival rate below 20% [1]. The dismal prognosis is mainly attributed to the high invasiveness and advanced stage at diagnosis. Despite the surgical resection offers a potentially curative treatment for a minority patients diagnosed at early stage, the incidences of loco-regional recurrence and distant metastases following surgery remain high. Understanding the molecular mechanisms of BTC progression in-depth is crucial for exploring early diagnostic and prognostic biomarkers, as well as effective therapeutic targets [2].

As crucial mechanisms in eukaryotic cells for temporal and spatial gene regulation, RNA modifications control gene expression and influence various physiological and pathological processes, including DNA damage response, stem cell differentiation, apoptosis, and embryonic development [3]. N6-Methyladenosine (m6A), a methylation modification at the N6 position of adenosine, is the most prevalent ample internal post-transcriptional modification on RNAs. M6A methylation modification is observed in almost all transcripts, with an average of 1–2 m6A residues per 1000 nucleotides [4, 5], mainly occurring on adenine in the consensus sequence RRACH (R = G or A; H = A, C, or U). Furthermore, about 70% of m6A are enriched in the coding region and 3’ untranslated region (3’UTR), while 20% occur in intron and 5’ untranslated region (5’UTR) [6, 7]. As a dynamic and reversible methylation process, m6A is involved in the regulation of different aspects of the RNA metabolism process, including mRNA splicing, translation, transportation, and degradation, and consists of “writer”, “reader”, and “eraser” [8]. Among them, deposition of methylation is catalyzed by m6A methyltransferase (writers) including methyltransferase-like 3 (METTL3), METTL14, Wilms’ tumor 1-associating protein (WTAP), RNA binding motif protein 15/15B (RBM15/15B), HAKAI, Zinc finger CCCH domain-containing protein 13 (Zc3h13), and VIRMA/KIAA1429. The removal of the modification is carried out by m6A demethylase (erasers) which include Fat mass and obesity associated protein (FTO) and alkB homolog 5 (ALKBH5), as well as ALKBH3. Dynamic regulation of distribution and abundance for m6A was maintained cooperatively by “writers” and “erasers”, while readers further recognize m6A-binding sites and mediate downstream fate of target RNAs, such as YT521-B homology (YTH) domain proteins, heterogeneous nuclear ribonucleoproteins C/G (HNRNPC/G), heterogeneous nuclear ribonucleoprotein A2B1 (HNRNPA2B1), insulin-like growth factor 2 mRNA-binding proteins 1–3 (IGF2BP1-3), and fragile X mental retardation protein (FMRP).

Recently, aberrant m6A modification has been found to play important in the tumorigenesis and development of tumors via influence the biological process of cell growth, differentiation, invasion, migration, drug resistance, and influencing the microenvironment. However, it is still controversy over whether different components of m6A regulators act as oncogenes or tumor suppressors in BTC. This review aims to provide a systematical overview of the advances in m6A modulation in BTC and their mechanisms and functions on RNA metabolism, which involved in the tumorigenesis and development of BTC, offering a rationale for the development of new biomarkers and epigenetic-based therapies.

## The molecular mechanism of m6A RNA modification

### m6A “writers”

Methyltransferase complex(MTC), composed of core components METTL3 and METTL14, are responsible for catalyzing the deposition of m6A modifications. As the main catalytic core of m6A methyltransferase complex, METTL3 linked with S-adenosylmethionine (SAM) transfers the methyl group to the adenosine base of RNA, thereby achieving methylation of target transcripts and regulating various physiological processes, including spermatogenesis [9], maintenance of T cell homeostasis [10], cell differentiation [11]. METTL14 was another methyltransferase and lacks catalytic activity, although also possesses its own methyltransferase domain. The METTL14-METTL3 heterodimer forms in nuclear speckles, and their synergistically enhance the catalytic activity and precise recognition of the MTC [12]. Recently, studies have shown that METTL14 could recognize histone H3 lysine 36 trimethylation (H3K36me3) modification and regulate the selective deposition of m6A in target mRNAs [13]. METTL16 is a methyltransferase for U6 spliceosomal small nuclear RNA (snRNA) and responsible for regulating the expression of the human MAT2A gene, which encodes most of the SAM synthase to maintain stable levels of SAM in cells [14]. WTAP is another key component of the m6A MTC, unlike METTL3 and METTL14, WTAP lacks the MTA methyltransferase domain and therefore has no methyltransferase activity. Instead, WTAP serves as a bridging protein to mediate RNA binding of MTC and nuclear localization of MTC [15]. RBM15/15B guides the METTL3-METTL14 heterodimer to the target mRNA by directly binding to U-rich sequences on the mRNA, which is essential for the process of m6A methylation [16]. VIRMA acts as a scaffold protein that recruits the METTL3/METTL14/WTAP complex to catalyze m6A methylation in the 3’UTR and near the stop codon of mRNA [17]. In addition, studies have shown that Zc3h13 serves as a new cofactor of the m6A MTC and maintains the nuclear localization of the Zc3h13-WTAP-Virilizer-Hakai complex, which promotes m6A methylation and regulates the self-renewal properties of mouse embryonic stem cells (mESCs) [18].

### m6A “erasers”

RNA m6A modification could be effectively removed in a non-heme Fe(II) and α-ketoglutarate dependent manner [19]. FTO, ALKBH5 and ALKBH3 were all identified as belonging to the ALKBH family with amino-terminal 2-oxoglutarate- and Fe(II)-dependent oxygenase domain [20–22], which can oxidative eliminate m6A residues in RNA. The change of demethylase activity will alter the methylation level of m6A in cells. FTO is the first m6A demethylase to be reported in human, localized in both the cytoplasm and nucleus. It mainly mediates demethylation of internal m6A and N6, 2’-O-dimethyladenosine in the 5’ cap of mRNA [23, 24], and regulates mRNA stability and alternative splicing [25]. FTO is widely expressed in fetal and adult tissues and mainly involved in energy metabolism in adipose and muscle tissues. ALKBH5 is the second identified m6A demethylase that is localized in nuclear speckles, and has been shown to regulate mRNA nuclear export and RNA metabolism by reducing the level of m6A in nuclear speckles [26]. ALKBH5 is highly expressed in testicular tissue and plays a crucial role in the compartmentalized regulation and splicing of 3’UTR-mRNA length during spermatogenesis. Meanwhile, it’s also involved in sperm development, embryonic development, autophagy, and innate antiviral response [27]. Differing from FTO and ALKBH5, ALKBH3 primarily recognizes m6A in tRNA rather than mRNA or rRNA, and the demethylation of tRNA mediated by ALKBH3 significantly enhances protein translation rate.

### m6A “readers”

**Fig. 1:**
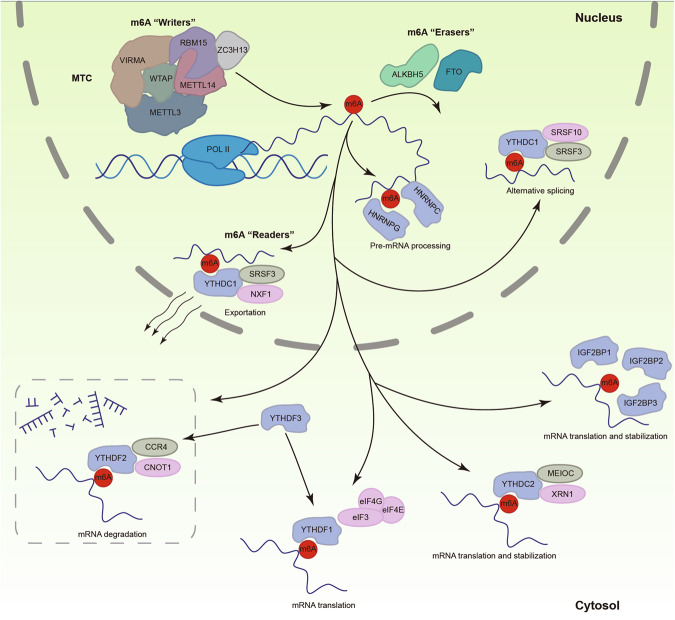
The molecular mechanism of m6A RNA modification. “Writers” catalyze the deposition of methylation. “Erasers” remove the m6A modification from RNA. “Readers” recognize m6A-binding sites and mediate RNA processing, splicing, exportion, translation, stabilization and decay.

The m6A writer and eraser maintain a dynamic balance between m6A deposition and removal. Readers, also called m6A-binding proteins, recognize and bind to the conserved RRm6ACH sequence on RNA transcripts and further effects target RNA metabolism and gene expression in multiple dimensions at the post-transcriptional level (Fig. 1). Readers mainly fall into three categories, YTH domain family, hnRNPs, and IGF2BPs, the RNA binding domain of which exhibited an 10-50 times higher affinity for m6A modified mRNA than unmodified mRNA [28].

#### Nuclear export and transcription of RNA

YTHDC1 mainly functions in the nucleus and participates in the regulation and maintenance of RNA transcription. Previous studies have shown that YTHDC1 can recruit the SET domain bifurcated histone lysine methyltransferase 1 (SETDB1) by recognizing a subset of retrotransposons -derived transcripts marked by m6A, to maintain the characteristics of mouse embryonic stem cells. Meanwhile, the interaction between YTHDC1 and SETDB1 inhibits the activity of retrotransposons and the two-cell (2 C) program inducer Dux [29]. In addition, YTHDC1 is involved in the transportation of mRNAs. Overexpression of YTHDC1 leads to a decrease of m6A mRNA levels in the nucleus, while downregulation of YTHDC1 results in the accumulation of m6A mRNA in the nucleus and a decrease in m6A levels in the cytoplasm. Mechanistically, after binding to m6A-methylated mRNA, YTHDC1 forms a mRNA export complex with SRSF3, NXF1, and TREX, and mediates export of m6A-modified mRNAs from the nucleus to the cytoplasm [30]. Besides YTHDC1, YTHDC2, as a 3′–5′ RNA helicase, also plays an important role in regulating mRNA transcription during meiosis [31].

#### Alternative splicing of pre-mRNA

The alternative splicing of mRNA precursors plays a crucial role in the regulation of transcript formation and functional diversity, and aberrant splicing is strongly associated with the development of various tumors. m6A modifications have been shown to orchestrate the interaction between trans- acting splicing factors and cis-regulatory RNA elements in eukaryotic cells [32]. YTHDC1 was identified to be localized in the Y bodies and modulate the splicing patterns of mRNA precursors in a concentration-dependent manner, which might be modulated by the interaction with pre-mRNA splicing factors SRSF3 and SRSF10 [33]. The competitive binding of SRSF3 and SRSF10 to the N-terminal domain of YTHDC1 results in differential splicing outcomes of downstream target mRNA. YTHDC1 with SRSF3 recognizes m6A-modified mRNA and promotes exon inclusion, while simultaneously inhibiting the binding of SRSF10 to mRNA, which can lead to exon skipping. The expression levels of YTHDC1 and SRSF3 have been reported associated with the development of various cancers, including breast cancer, colorectal cancer, ovarian cancer, osteosarcoma, gliolastoma, and prostate cancer. Increasing evidence revealed the pioctal role of YTHDC1 in cancer through its involvement in alternative splicing of mRNA precursors [34, 35].

#### Translation of mRNA

M6A readers YTHDF1, YTHDF2, YTHDF3, and YTHDC2 are all considered to be involved in the regulation of mRNA translation. YTHDF1 directly binds to the m6A sites nearby the stop codon and recruits eukaryotic translation initiation factors (eIF) 3, 4E, 4 G, and poly(A)-binding protein (PABP), which consequently facilitates ribosome assembly and promotes the process of mRNA cap-independent translation, rather than classical cap-dependent translation [36–38]. Previous findings suggested that YTHDF3 shares mRNA targets with YTHDF1 and facilitates its translation efficiency, by recruiting translation machineries eIFs directly, or interacting with YTHDF1 and affect the function of YTHDF1 on translation [39, 40]. YTHDF2 has also been identified as an independent translation regulatory factor for m6A-modified mRNA, promoting tumor growth by facilitating the translation of 6PGD mRNA. In addition, YTHDC2 interacts with the 5′–3′ exoribonuclease XRN1, and exerts its positive regulatory effect on translation independently through its RNA helicase activity, which enables the unwinding and resolving of mRNA secondary structures and subsequently enhances translation elongation [41].

#### mRNA stability and mRNA decay

Among several m6A readers, YTHDF2 is recognized as RNA binding protein primarily promoting decay of targeted mRNA [42]. YTHDF2 has been shown to result in m6A mRNA degradation via two distinct pathways: (i) deadenylation-dependent decay, which initiated by shortening of the 3′-poly(A) tail via YTHDF2-CCR4 / NOT axis and (ii) the endonuclease dependent decay pathway mediated by the YTHDF2-HRSP12-RNase P/MRP axis. In the YTHDF2-CCR4 / NOT axis pathway, YTHDF2 directly recognizes m6A modification through its C-terminal domain and interacts with the superfamily homologous (SH) domain of CNOT1 at its N-terminus, subsequently promoting RNA deadenylation and degradation mediated by the CCR4/NOT deadenylase complex, and facilitating the formation of P bodies [43]. On the other hand, for the YTHDF2-HRSP12-RNase P/MRP axis pathway, HRSP12 is recruited to YTHDF2 and simultaneously binds to the upstream site of m6A in target mRNA. YTHDF2 then recruits RNase P/MRP endoribonucleases, which cleave the target mRNA at the downstream binding site, thereby degrading m6A mRNA [44]. In addition, YTHDF3, another cytoplasmic m6A binder of the YTH domain family, has been indicated to affect the target binding activity and specificity of YTHDF2. Shi et al. showed that YTHDF3 and YTHDF1 cooperatively facilitate partitioning of m6A marked RNA to YTHDF2 for accelerated decay [40].

Contrary to the role of YTHDF2 in promoting mRNA degradation, IGF2BP s as a newly identified family of m6A readers, has been reported to enhance mRNA stability. This effect is likely attributed to its competitive binding with YTHDF2 and its cofactors, HuR and MATR3 [45]. HuR interacts with IGF2BPs and colocalized in P-bodies, which can protect m6A-containing mRNA from degradation and block microRNA targeting [46]. IGF2BPs have also been found to promote translation during heat shock response. Furthermore, YTHDF1 stabilizes c-Myc mRNA through an m6A-dependent pathway, thereby promoting the development of oral squamous cell carcinoma [47]. Data from Hiroki Shima et al. has revealed that YTHDC1 may also play a role in regulating the mRNA stability of MAT2A, although the specific molecular mechanisms are yet to be elucidated [48].

## Function of m6A in BTC

**Fig. 2:**
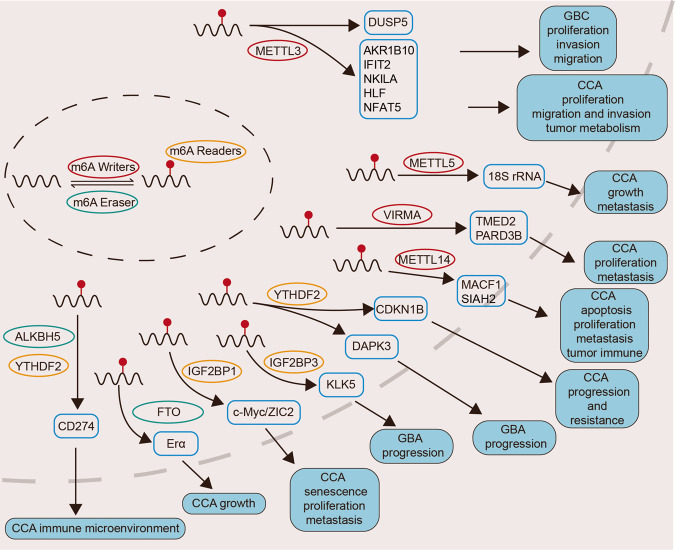
Function of m6A in BTCs. m6A regulators act as oncogenes or tumor suppressors through m6A depended regulation of the target RNAs in BTCs.

Studies have shown that the abundance of m6A modification and the expression levels of m6A-related genes (writers, erasers and readers) are frequently dysregulated in various types of malignant tumors [49], which are critically involved in the development, proliferation [50], metastasis, invasion [51], drug resistance [52, 53], and immune evasion of cancer [54, 55]. For example, recent researches have suggested that the level of m6A methylation can regulate the activation of PI3K-Akt-mTOR pathway and participate in tumor glucose metabolism [56]. Nie et al. demonstrated that demethylase ALKBH5 was significantly upregulated in cisplatin-resistant ovarian cancer and activated JAK2/STAT3 pathway by mediating JAK2 m6A demethylation to promote the resistance to cisplatin [57]. METTL3 also was identified an important oncogenic role in the invasion and gemcitabine resistance of pancreatic ductal adenocarcinoma, via regulating metabolism of DDX23 mRNA [58]. With the maturation of methylated RNA immunoprecipitation (MeRIP) and next-generation sequencing technologies, the pivotal roles and molecular mechanisms of an increasing number of m6A-related genes in BTCs have been elucidated (Fig. 2). Data from The Cancer Genome Atlas (TCGA) have also shown that several m6A-related genes have an impact on the prognosis of CCA patients and are associated with immune infiltration and response to immunotherapy in CCA patients [59]. We summarize the current studies on the regulatory effects of m6A methylation modification in BTCs and list in the Table 1.

**Table 1:** The role of m6A regulators in BTCs.

m6a regulators	Type of BTC	Type of regulation	Target	Mode of RNA metabolism	Functions	Ref.
METTL14	CCA (iCCA and pCCA)	suppressor	MACF1/β-catenin	Stabilization	Promotes CCA apoptosis and inhibits CCA proliferation and metastasis.	[61]
METTL3	GBC	oncogene	DUSP5	Degradation	Premotes GBC proliferation, invasion, and migration.	[65]
METTL3	CCA (iCCA)	oncogene	AKR1B10	Stability	Promotes CCA proliferation, invasion, glucose uptake, and lactate production.	[66]
	CCA (iCCA)	oncogene	IFIT2	Degradation	promotes ICC cell proliferation, invasion, and migration.	[69]
	CCA (iCCA)	oncogene	HLF	Stabilization	Promotes ICC self-renewal, tumorigenicity, proliferation and metastasis	[67]
	CCA (iCCA)	oncogene	NFAT5	Stability	Promotes ICC aerobic glycolysis, proliferation, metastasis	[68]
METTL3	CCA (eCCA)	oncogene	NKILA/miR-582-3p/YAP1	Interaction between NKILA and miR-582-3p	Promotes CCA growth and metastasis.	[71]
METTL5	CCA (iCCA)	oncogene	18S rRNA	Hinder ribosome synthesis	Promotes ICC growth and metastasis	[70]
VIRMA	CCA (iCCA)	oncogene	TMED2, PARD3B	Stabilization	Promotes ICC proliferation and metastasis	[90]
YTHDF2	CCA (iCCA)	oncogene	CDKN1B	Degradation	Promotes ICC progression and cisplatin insensitivity.	[72]
YTHDF2	GBC	oncogene	DAPK3	Degradation	Promotes GBC progression.	[73]
YTHDF1	CCA (iCCA)	oncogene	EGFR	Translation	Promotes ICC cell growth and aggressive abilities.	[74]
IGF2BP3	GBC	oncogene	KLK5/PAR2/Akt1	Stability	Promotes GBC progression.	[75]
IGF2BP1	CCA (iCCA)	oncogene	c-Myc/p16	Stabilization	Promotes iCCA progression.	[76]
	CCA (iCCA)	oncogene	ZIC2/PAK4/AKT/MMP2	Stability		
FTO	CCA (iCCA and eCCA)	suppressor	TEAD2	Stability	Promotes CCA apoptosis, inhibits anchorage-independent growth and mobility.	[77]
	CCA (iCCA and eCCA)	oncogene	ERα/miR16-5p/YAP1	Translation	Promotes CCA cell growth.	[78]
ALKBH5	CCA(iCCA)	sensitize anti-PD1 immunotherapy	CD274	Degradation	Promotes the expression of PD-L1 and the sensitivity to PD-1 immune therapy.	[83]
METTL14	CCA(iCCA)	sensitize anti-PD1 immunotherapy	SIAH2/PD-L1	Degradation	Inhibits T cells expansion and cytotoxicity by sustaining tumor cell PD-L1 expression.	[84]

BTC, Biliary tract cancer; CCA, Cholangiocarcinoma; iCCA, Intrahepatic cholangiocarcinoma; pCCA, Perihilar cholangiocarcinoma; GBC, Gallbladder cancer; eCCA, extrahepatic cholangiocarcinoma.

### Function of m6A “writers” in BTC

The genomic alterations in different anatomical subtypes of BTCs show significant heterogeneity [60]. Zhang et al. conducted whole-exome sequencing analysis on 348 samples of BTCs from three distinct subtypes, providing insights into the genomic landscape of perihilar cholangiocarcinoma (pCCA) and iCCA. Several m6A related genes have been identified as key drivers in CCA development, and the common mutational patterns have been fully characterized [61]. Notably, methyltransferase METTL14 and demethylase RBM10 were identified as significantly mutated genes (SMGs) in CCA, particularly with multiple high-frequency non-synonymous mutations observed in pCCA, leading to functional loss of the genes [62–64]. Furthermore, multiple mutation sites of METTL14 gene in CCA are specifically directed toward the 298 amino acid residue, which is a critical locus for the RNA binding with METTL3-METTL14 complex [12]. It has been demonstrated that the low expression and functional disruption of METTL14 in CCA result in a decrease in mRNA methylation levels of MACF1, subsequently inhibiting its degradation [61]. MACF1, known as an oncogene, is involved in the regulation of the WNT signaling pathway and tumor epithelial-mesenchymal transition (EMT). Studies have revealed that METTL14 catalyze the formation of m6A modification on MACF1 and regulates the proliferation, apoptosis, and expression of EMT-related proteins in CCA both in vitro and in vivo.

Recently, the role of another methyltransferase METTL3 has also been identified in GBC, which is associated with higher clinical stage and poor prognosis in GBC cases. Functionally, METTL3 enhances the degradation of DUSP5 mRNA in a YTHDF2-dependent manner, thereby contributing to the increase of proliferation, invasion, and metastasis of NOZ and GBC-SD cells [65]. In CCA, METTL3 has been indicated to upregulate the expression of oncogenes such as AKR1B10, HLF and NFAT5, which consequently promote growth, self-renewal, metastasis and glycolysis of ICC cells [66, 67], and is proven to be correlate with poor prognostics [68]. Interferon-induced protein with tetratricopeptide repeats 2 (IFIT2) has also been identified as a target of METTL3 in cholangiocarcinoma. However, contrastly, the degradation of IFIT2, rather than its stability, is stimulated by METTL3 in a YTHDF2-dependent manner [69]. Besides mRNA, m6A “writers” have also been found involved in the m6A modification in non-coding RNAs. For instance, METTL5-mediated 18 S rRNA m6A modification hinders ribosome synthesis to inhibit the translation of G-quadrupler-containing mRNAs and the TGF-β signal pathway [70]. METTL3 enhances the adsorbing of the long non-coding RNA NKILA with miR-582-3p in a m6A dependent manner, which thereby upregulates the expression of YAP1 and contributes to CCA progression [71].

### Function of m6A “readers” in BTC

YTHDF1 and YTHDF2 have been found to be upregulated in ICC tissues, which is often associated with poor prognosis. Notably, significant overexpression of YTHDF2 has been verified in cisplatin-resistant ICC tissues, and been demonstrated to promote cell proliferation, inhibit apoptosis, reduce G0/G1 phase arrest, and confer resistance to cisplatin in ICC cells. The oncogenic role of YTHDF2 is attributed to its recognition to the m6A site GGACA in the 3’ UTR region of CDKN1B mRNA, which facilitates its decay process and consequently suppresses DNA damage in ICC cells [72]. These findings provide novel opportunities for improving the clinical efficacy of cisplatin chemotherapy in ICC patients [71]. In addition, YTHDF2 was also identified to influence the progression of GBC by facilitated the degradation of the mRNA of tumor suppressor DAPK3, in which the CCR4/NOT deadenylase complex was involved [73]. YTHDF1, on the other hand, enhances the translation of epidermal growth factor receptor (EGFR) mRNA in ICC. Research by Xiang Huang et al. demonstrated that upregulation of YTHDF1 significantly enhances the protein expression of EGFR in ICC cells, rather than at the mRNA level [74]. Another m6A reader IGF2BP3 has also been shown to function as an oncogene in GBC, which regulates the proliferation and metastasis both in vivo and in vitro. The underlying molecular mechanism might be mediated through the stability enhancement of KLK5 mRNA and the activation of the KLK5/PAR2/Akt1 axis. Additionally, it has been observed that the regulation of this process may be influenced by cancer-associated miRNA let-7g-5p [75]. Previous study validated that IGF2BP1 was the crucial protein in stabilizing the RNA of c-Myc and ZIC2 in CCA, thereby promoting iCCA growth and suppressing its senescence through the c-Myc/p16 axis, while inducing metastasis via activating the ZIC2/PAK4/AKT/MMP2 axis. Importantly, the application of IGF2BP1 inhibitors have demonstrated promising anti-cancer efficacy in patient-derived xenograft (PDX) mouse models, and provide a novel targeted therapeutic strategies for CCA [76].

### Function of m6A “erasers” in BTC

FTO, the first identified m6A methyltransferase, plays a crucial role in controlling fatty acid transport, synthesis, metabolism, and susceptibility to obesity in eukaryotic cells. The roles of FTO in different types of tumors are also heterogeneous. It has been reported that FTO expression is downregulated in ICC tissues and cell lines, which is negatively correlated with the micro-vessel density (MVD) and CA19-9 levels. Zhuo-Xian Rong et al. indicated that FTO disrupts the stability of the oncogene TEAD2 mRNA, suppressing various malignant behaviors of ICC, and is significantly associated with clinical prognosis [77]. Furthermore, the protein translation process of estrogen receptor alpha (ERα), another target of FTO, is suppressed due to the increased m6A levels on its mRNA, consequently inhibiting CCA tumor growth [78].

## M6A modification in the immune microenvironment of BTC

Besides its direct influence on tumor cells, m6A methylation has been found to modulate tumor development through its involvement in tumor immune microenvironment. For instance, it has been demonstrated that the transcription and expression of m6A-marked mRNAs encoding lysosomal proteases were enhanced via m6A-dependent pathways, which subsequently facilitate the degradation of tumor antigens engulfed by immune cells. As a result, the antigen cross-presentation by dendritic cells (DCs) was suppressed and immune evasion was induced [79]. M6a modification also occurs on immune molecules, which thereby affects their activity and subsequently regulates the migration, metabolism, differentiation, and maturation of immune cells [10, 80, 81]. Previous studies have demonstrated that the differential m6a modification patterns in BTC tumor tissue are associated with the heterogeneity of the tumor microenvironment and the level of immune cell infiltration [82]. In cholangiocarcinoma, the high expression of m6A-associated genes promotes the infiltration of memory B cells, immature B cells, plasma cells, Tregs, monocytes, resting dendritic cells, and eosinophils. In this regard, the high expression of CRBPB, SDC1, VPS25, and AIP facilitates the process of Treg cells and resting DC cells infiltrating to cholangiocarcinoma tissue and is associated with the poor prognosis in ICC patients [59].

Qiu et al. recently reported the immunomodulatory role of the demethylase ALKBH5 in intrahepatic cholangiocarcinoma’s (iCCA) immune microenvironment [83], which showed that PD-L1 was subject to post-transcriptional regulation mediated by m6A modification. Knockdown of ALKBH5 increased the enrichment of m6A nearby the stop codon and 3’UTR region of CD274 mRNA, thereby promoting the decay of CD274 mRNA in a YTHDF2-dependent manner, but ALKBH5 has minimal inhibitory effects on tumor cell PD-L1 expression at the translational level. On one hand, ALKBH5 can modulate the expression of PD-L1, suppressing the proliferation of CD3 + T cells and the cytotoxicity of CD8 + T cells in tumor microenvironment. On the other hand, single-cell mass spectrometry analysis revealed that ALKBH5 can enhance the expression of PD-L1 in monocytes/macrophages within the ICC microenvironment while inhibiting the infiltration of myeloid-derived cells. Notably, ICC patients with a high expression pattern of ALKBH5 tend to exhibit enhanced sensitivity to PD-1 immune therapy. Zheng et al. demonstrated the role of the METTL14-Siah2-PD-L1 axis in immunotherapy for cholangiocarcinoma [84]. METTL14, through its m6A modification on the 3’ end of E3 ubiquitin ligase Siah2 mRNA, has been shown to promote its YTHDF2-dependent degradation. As an E3 ubiquitin ligase specifically recognizing PD-L1, the downregulation of Siah2 facilitates the expression of PD-L1, thereby enhancing sensitivity to anti-PD-1 immune therapy.

## Aberrant regulation of m6A in BTC

**Fig. 3:**
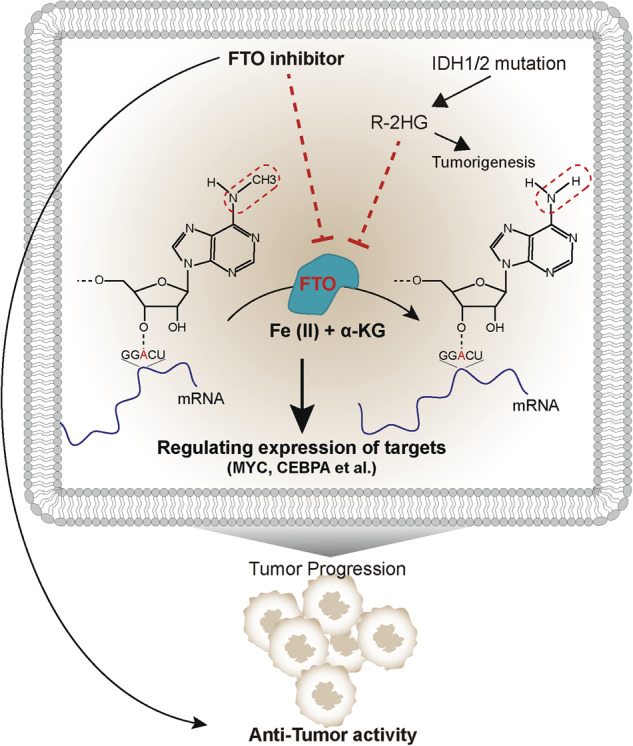
The anti-tumor effects of FTO inhibitor. FTO inhibitor acts anti-tumor activity in an m6A-depended manner, especially in cases with IDH1/2 mutation. R-2HG promotes tumorigenesis while also increasing abundance of m6A modifications through inhibiting FTO, and exhibiting anti-tumor effects.

The expression of m6A-related genes is dysregulated by a variety of mechanisms. In the tumorigenesis and development of CCA, it is well-known that inflammation plays a pivotal role. Notably, the expression levels of m6A writers METTL3, METTL14, and WTAP have been found to be regulated by inflammatory factors present in the CCA tumor microenvironment. IL-6 triggers inflammatory response and activates STAT3, resulting in the upregulation of m6A writers’ transcription. As a consequence, the stability and translation of CTNNB1 and CD133 are enhanced, thereby promoting the stemness of CCA cells [85]. IDH1/2 mutations, one of the most characteristic gene mutations in cholangiocarcinoma, result in elevated levels of (R)-2-hydroxyglutarate (R-2HG), which inhibits the α-ketoglutarate (α-KG)-dependent dioxygenase activity of FTO (Fig. 3). This also leads to an increase in m6A abundance [78, 86]. Consequently, the elevated methylation level of ERα inhibits its binding to ribosomes and the process of protein translation (rather than YTHDF2-mediated mRNA degradation), and further suppresses CCA growth via the ERα/miR16-5p/YAP1 signaling pathway. Additionally, the liver produces deoxycholic acid (DCA), which is stored in the gallbladder and functions as a metabolic molecule with multiple endocrine and paracrine activities. It is reported that DCA has been found significantly downregulated in gallbladder cancer and acts as a tumor suppressor factor [87, 88]. Mechanistically, DCA in gallbladder cancer promotes the dissociation of METTL3 from the MTC (METTL3-METTL14-WTAP) complex, which inhibits the miR-92b-3p expression in an m6A-dependent manner. This process relieves the inhibitory effect of miR-92b-3p on its target PTEN, which suppresses the PI3K/AKT signaling pathway to play a role of tumor suppressor gene in GBC. In another study, it was found that the cytokine CCL3 secreted by liver normal cells regulates the m6A methylation level in ICC. Through the interaction with CCR5, CCL3 upregulates the expression of VIRMA in ICC. VIRMA further modulates the catalytic activity of MTC, which enhances the methylation modification and expression of SIRT1, promoting the tumor cell proliferation and migration of ICC [89]. In addition, the promoter region demethylation of H3K27me3 modification in ICC also contributes to the upregulation of expression of VIRMA, and subsequently promotes ICC proliferation and metastasis via TMED2/Akt/GSK/β-catenin and PARD3B/ MEK/ERK/Slug pathways [90].

## Therapeutic strategies based on m6A modification

The development of drug resistance represents a significant challenge in BTC, leading to poor clinical outcomes. Modulating RNA m6A modification has been suggested to be a promising strategy in various types of cancer. In addition to regulating tumor-related signaling pathways and the tumor microenvironment, m6A RNA modification has been found to intervene in the resistance of tumor cells to drug treatment by modulating various drug efflux protein transporters (such as ABCG2, ABCC9, ABCC10), drug-metabolizing enzymes (such as CYP2C8), and drug targets (such as p53 R273H), among others [91]. Currently, several small molecules targeting m6A regulators have been developed, most of which focus on FTO inhibitors [92].

As aforementioned, FTO, as an enzyme belonging to the Fe2+ and α-ketoglutarate-dependent AlkB dioxygenase family (2OGX), plays a critical role in various biological processes involved in tumor initiation and progression. Furthermore, epidemiological studies have indicated a significant increase in the incidence of multiple cancers in individuals with high FTO expression and obesity [93]. Small-molecule inhibitors targeting FTO have been considered to have potential therapeutic effects in the field of cancer treatment. FTO catalyzing the demethylation reaction requires 2-oxoglutarate (2OG) and Fe(II) as cofactors. Thereby, R-2HG, produced by IDH1/2 mutations, functions as a 2OG analog and can competitively inhibits the activation of FTO (Fig. 3). However, in order to avoid inhibiting unexpected 2OGXs and undesirable side effects, achieving specificity in FTO recognition has become the key in drug development and treatment [91]. Entacapone and meclofenamate (MA) 2, approved by the U.S. Food and Drug Administration (FDA) as FTO inhibitors, have been demonstrated to hold the efficacy in improving chemotherapy drug sensitivity and inhibiting tumor progression in multiple cancer types [94, 95]. Despite the anti-tumor effects of FTO inhibition have shown great promise in cholangiocarcinoma, there is currently a lack of data to support its application in in vivo models and clinical settings. However, recent studies have identified new and promising FTO inhibitors as shown in Table 2 [95–114], including FB23, FB23-2, CS1, and CS2, which have exhibited potent anti-tumor effects with minimal side effects in the treatment of acute myeloid leukemia (AML) [101, 107]. This highlights their significant clinical value. What’s more, the exploration of novel effective therapeutic strategies targeting BTC holds great promise through the use of bioengineered fusion proteins containing dCas13 for targeted RNA demethylation or methylation [115–117].

**Table 2:** Antitumor effects of known FTO inhibitors.

FTO inhibitor	Competitive binding site	anti-cancer activities	ref.
R-2HG	2OG binding site	Inhibit proliferation/survival of leukemia via FTO/m6A/MYC/CEBPA signaling in leukemic cells without IDH mutations.	[96]
meclofenamic acid (MA) and MA2	substrate binding site	Inhibit the survival, growth and self-renewal of GBM cells.	[97]
		Enhance the effect of temozolomide on suppressing proliferation of glioma cells.	[98]
		Inhibit brain metastasis of breast cancer cells.	[99]
		Increase Gefitinib (GE) Sensitivity of GE-resistant NSCLC cells via FTO/m6A-Demethylation/c-Myc pathway.	[100]
MA derivatives: FB23 and FB23-2	substrate binding site	Suppress the proliferation and promote the differentiation and apoptosis of AML cells.	[101, 102]
Dac51	substrate binding site	Increase the infiltration and cytotoxic capacity of T cells.	[102]
		Inhibit the growth of melanoma and synergize with anti-PD-L1 therapy.	[103]
FTO-4	substrate binding site	Inhibit neurosphere formation ability of GSCs.	[95]
FTO inhibitor 18097	substrate binding site	Inhibit cellular lipogenesis, suppress growth and lung colonization of breast cancer cells, by P53 signaling pathway and PPARγ/C/EBPα/C/EBPβ pathway.	[104]
Entacapone	Substrate- and 2OG-binding site	Inhibited the proliferation and colony formation ability of osteosarcoma cells via FTO/m6A/DACT1 axis.	[105]
MO-I-500	-	Inhibit the survival and colony formation of SUM149.	[106]
CS1 (bisantrene) and CS2 (brequinar)	-	Inhibite the activity of leukemia stem the immune escape of leukemia cells.	[107]
Compound C6	-	Inhibit esophageal cancer cells proliferation by EMT pathway and PI3K/AKT pathway.	[108]
Rhein	substrate binding site	Unkonwn	[109]
Fluorescein 1	substrate binding site	Unkonwn	[110]
Radicicol	substrate binding site	Unkonwn	[111]
compound 12	Substrate- and 2OG-binding site	Unkonwn	[112]
Nafamostat mesilate	-	Unkonwn	[113]
Diacerein	-	Unkonwn	[114]

## Conclusions and outlook

Collectively, m6A, as a significant mechanism of post-transcriptional modification, exerts crucial regulatory effects on the occurrence and progression of BTC. Dysregulated expression of m6A regulatory genes in BTC disrupts the normal expression of downstream RNAs and proteins, resulting in the activation or inhibition of cancer-related signaling pathways. Consequently, various phenotypic characteristics such as proliferation, invasion, metastasis, apoptosis, drug resistance, and tumor immune responses are influenced in BTC. Moreover, the expression patterns of m6A-related genes can serve as predictive biomarkers for BTC diagnosis, prognosis, and drug treatment response. However, the distinct mechanisms and differ roles of m6A modifications in different stages and sites of BTC need to be elucidated. The extent of crosstalk among different m6A regulators is largely unknown. Additionally, rigorous clinical trials are necessary to evaluate the efficacy and safety of m6A-targeting small molecule inhibitors and other therapeutic approaches in the management of BTC. Further exploration and characterization of the targets and molecular mechanisms underlying m6A modification in BTC are warranted to develop effective strategies for targeted therapy in future BTC patients.

## Data Availability

The studies included were retrieved from the PubMed database.
